# Permanent Whisker Removal Reduces the Density of c-Fos+ Cells and the Expression of Calbindin Protein, Disrupts Hippocampal Neurogenesis and Affects Spatial-Memory-Related Tasks

**DOI:** 10.3389/fncel.2018.00132

**Published:** 2018-05-15

**Authors:** Oscar Gonzalez-Perez, Verónica López-Virgen, Nereida Ibarra-Castaneda

**Affiliations:** ^1^Laboratory of Neuroscience, School of Psychology, University of Colima, Colima, Mexico; ^2^El Colegio de Colima, Colima, Mexico; ^3^Medical Sciences PhD Program, School of Medicine, University of Colima, Colima, Mexico

**Keywords:** subgranular zone, adult neurogenesis, whisker, tactile system, calbindin, hippocampus, memory, dentate gyrus

## Abstract

Facial vibrissae, commonly known as whiskers, are the main sensitive tactile system in rodents. Whisker stimulation triggers neuronal activity that promotes neural plasticity in the barrel cortex (BC) and helps create spatial maps in the adult hippocampus. Moreover, activity-dependent inputs and calcium homeostasis modulate adult neurogenesis. Therefore, the neuronal activity of the BC possibly regulates hippocampal functions and neurogenesis. To assess whether tactile information from facial whiskers may modulate hippocampal functions and neurogenesis, we permanently eliminated whiskers in CD1 male mice and analyzed the effects in cellular composition, molecular expression and memory processing in the adult hippocampus. Our data indicated that the permanent deprivation of whiskers reduced in 4-fold the density of c-Fos+ cells (a calcium-dependent immediate early gene) in *cornu ammonis* subfields (CA1, CA2 and CA3) and 4.5-fold the dentate gyrus (DG). A significant reduction in the expression of calcium-binding proteincalbindin-D_28k_ was also observed in granule cells of the DG. Notably, these changes coincided with an increase in apoptosis and a decrease in the proliferation of neural precursor cells in the DG, which ultimately reduced the number of Bromodeoxyuridine (BrdU)+NeuN+ mature neurons generated after whisker elimination. These abnormalities in the hippocampus were associated with a significant impairment of spatial memory and navigation skills. This is the first evidence indicating that tactile inputs from vibrissal follicles strongly modify the expression of c-Fos and calbindin in the DG, disrupt different aspects of hippocampal neurogenesis, and support the notion that spatial memory and navigation skills strongly require tactile information in the hippocampus.

## Introduction

Facial vibrissae, also referred to as whiskers, are the main sensitive tactile system in rodents. Each vibrissal follicle has a neuronal representation in the barrel cortex (BC), in layer IV of the somatosensory cortex (Woolsey and Van der Loos, 1970). Whisker stimulation increases the neuronal activity that, in turn, produces functional changes and neural plasticity in the BC (Lecrux et al., 2011). Some studies have shown that the whisker tactile information linked to fine discrimination increases the spiking rate in the CA1 hippocampal region (Pereira et al., 2007; Itskov et al., 2011). In fact, hippocampal place cells integrate tactile inputs from vibrissal follicles (Gener et al., 2013). Whisker elimination during early brain development reduces the activity of CA3 neurons, which induces CA3-CA1 synaptic facilitation (Milshtein-Parush et al., 2017). This evidence indicates that many of the tactile inputs from the vibrissal system are processed in the hippocampus, and this tactile information seems to play an important role in the creation of spatial maps (Pereira et al., 2007).

The CA1 hippocampal region contains pyramidal neurons that create spatial maps by integrating sensorial information (Muller and Kubie, 1987). Neuronal activity in CA1 is also modified by the electrical activity of the new neurons generated in the dentate gyrus (DG; Kitamura and Inokuchi, 2014). In the adult brain, the subgranular zone (SGZ) of DG produces new neurons that migrate shortly into the granular layer and spread their axons to CA3 (Kempermann et al., 2015). To date, it is well accepted that hippocampal neurogenesis modulates the processes of memory acquisition and retention (Aimone et al., 2006). Inputs from voluntary exercise, enriched environments and cognitive processes comprise a group of stimuli that control hippocampal functions in an activity-dependent manner that, in turn, modulates the adult neurogenesis (Kempermann et al., 1997; Pereira et al., 2007; Ma et al., 2009). Therefore, there is an exciting possibility that whisker-derived information modulates the neurogenic process in the DG. To assess this theory, we analyzed the effect of permanent whisker deprivation (WD) on the neuronal activity of hippocampal regions, adult neurogenesis in the DG and hippocampal functions. Our findings indicate that the permanent deprivation of whiskers strongly reduces the density of c-Fos+ cells (a calcium-dependent immediate early gene) and calbindin (a calcium-binding protein) in several hippocampal regions, including DG. WD is also associated with a dramatic decrease in several aspects of adult neurogenesis and produces a significant impairment of spatial memory and navigation. These data support the notion that tactile inputs are crucial to maintain the adult neurogenesis, create spatial maps in the hippocampus, and preserve memory performance.

## Materials and Methods

### Animals

Eight-week-old male CD-1 mice were maintained at a constant room temperature (22 ± 2°C), 12-h light/dark cycle, with *ad libitum* access to food and water. All rodents were randomly assigned to experimental and control groups that were kept under the same biotery and handling conditions throughout the study. All procedures were in accordance with the Principles of Laboratory Animal Care (Mexican Official Norm [NOM] 062-ZOO-1999) and approved by the University of Colima, Animal Care Committee.

### Whisker Deprivation (WD)

At the postnatal 60 (P60) day, the whisker-deprived group was intraperitoneally anesthetized with ketamine (10 mg/kg) plus xylazine (5 mg/kg) and the facial vibrissae were fulgurated under a surgical microscope (OPMI Vario/S88, Carl Zeiss Germany). Briefly, each whisker was gently pulled out and ablated with a surgical cautery device. The control group received the same pharmacological manipulation, but no whisker removal procedure was done. After that, the animals were placed in a pre-warmed cage until recovery. We quantified the bodyweight gain every 2 days and corroborated the fact that facial injuries healed in less than 72 h.

### Bromodeoxyuridine (BrdU) Administration

Bromodeoxyuridine (BrdU) injections were used for two different experiments. To evaluate the short-term cell division, the animals (*n* = 5 per group) received an intraperitoneal injection of BrdU (50 mg/kg; Sigma B5002, St. Louis, MO, USA) dissolved in 0.007 N NaOH solution, 2 h before sacrifice (Xie et al., 2016). To label the cell proliferation at the long term, the mice (*n* = 5 per group) received an intraperitoneal injection of 50 mg/kg BrdU every 8 h for the first 3 days (Gonzalez-Perez et al., 2011). These BrdU injections were done 72 h after vibrissal fulguration to minimize the effect of inflammatory cytokines triggered by the surgical procedure.

### Memory Test

Spatial memory was tested in the Barnes Maze (Barnes, 1979). The apparatus consisted of a circular platform (90-cm diameter) with 12 equally spaced holes (5.5-cm diameter) located at one centimeter from the edge of the platform. Visual cues consisted in four different geometrical pieces placed around the maze. Only one hole had access to the shelter hole, a black container (23 cm × 6 cm × 6 cm) attached under the platform. Twenty-four days after whisker elimination, the animals were covered with a black cylinder (the starting box), placed at the center of the maze and allowed to freely move onto the platform. The test consisted of three phases: habituation, acquisition and retention. For the habituation phase (experimental day 24), the mice were exposed to the components of the apparatus, platform, goal and starting box for 2 min, which helps reduce the anxiety induced by a novel environment. In the acquisition phase, the animal was placed at the center of the platform inside of the starting box for 15 s and allowed to move freely for 4 min. If the animal did not find the shelter hole in 4 min, it was guided and placed manually in the goal box for 1 min. After that, the mouse was returned to its home cage for 2 min, while the apparatus was cleaned with 70% ethanol solution to eliminate odor cues. This procedure was repeated four times per day for 3 days. For the retention phase or probe trial (48 h after the last trial), the shelter box was removed, and mice were allowed to explore the maze for 4 min. For the acquisition trials, latency was defined as the time spent for the animal to enter the goal box. Instead, latency in the retention phase was defined as the time needed to reach the goal hole. We recorded the number of errors to the first encounter of the escape hole (primary errors) and path length for the entire duration of assays (total path length). The search strategy employed for the animals to solve the memory test during the acquisition phase was classified as: random, serial or spatial (Jašarević et al., 2011; Williams et al., 2013). The random strategy was characterized by multiple central crosses in the maze and random searching behavior. The serial strategy was defined as systematic searching behavior in consecutive holes and with maximum two central crosses. The spatial strategy was identified as navigation directed to the goal box without crossing the center of the platform more than once. All trials were recorded with a digital camera and analyzed using the EthoVision tracking system (Noldus Equipment, Wageningen, Netherlands).

### Tissue Preparation

Animals were sacrificed with 100 mg/kg pentobarbital i.p. and perfused transcardially with 25 ml of 0.9% NaCl solution followed by 25 ml of 4% paraformaldehyde in 0.1 M phosphate buffer solution (PB), pH 7.4. The brains were extracted and post-fixed overnight in 50 ml of the same fixative solution. Then, we cut 20-μm coronal sections serially from −0.82 mm to −3.70 mm coordinates relative to Bregma (Paxinos and Watson, 2007) using a vibratome (Leica VT100S, Nussloch, Germany).

### Cytochrome Oxidase Histochemistry

Cytochrome oxidase allows labeling the BC (Wong-Riley and Welt, 1980). Brain tissues were washed three times in 0.1 M PB saline solution (PBS) and incubated in cytochrome oxidase (0.5 mg sucrose, 0.6 mg diaminobenzidine (DAB) and 30 mg Cytochrome C; all from Sigma-Aldrich) in 10 ml of 0.2 M PB (pH = 7.4) for 4 h at 37°C. The samples were washed in 0.1 M PBS, mounted on glass slides, dehydrated and sealed with resin (Entellan, Millipore).

### Immunohistochemistry

Brain sections were rinsed three times in 0.1 M PBS. To inactivate endogenous peroxidases, the sections were incubated in 3% hydrogen peroxide (H_2_O_2_) for 30 min and then washed three times (3×) in 0.1 M PBS. The samples were incubated in blocking solution (0.1 M PBS, 10% fetal bovine serum and 0.1% Triton X-100) for 40 min. Subsequently, the samples were incubated with the primary antibody rabbit anti-c-Fos (dilution 1:800; Cell Signaling Technology Cat # 2250, RRID:AB_2247211) and rabbit anti-Calbindin-D_28k_ (dilution 1:1000; Synaptic Systems Cat # 214002, RRID:AB_2068199) dissolved in blocking solution at 4°C overnight. The next day, the brain sections were rinsed 3× in 0.1 M PBS and incubated with the biotinylated secondary antibody (goat anti-rabbit IgG + IgM Biotin, dilution 1:200; Sigma-Aldrich; St. Louis, MO, USA) in 0.1 M PBS and 10% fetal bovine serum, for 1 h. Then, the samples were washed 3× and incubated with avidin-biotin complex (Vectastain Elite ABC kit, Vector Laboratories, Burlingame, CA, USA) for 60 min and rinsed in 0.1 M PBS. The sections were revealed for 5 min with 0.03% DAB solution (Sigma-Aldrich) plus 0.05% nickel ammonium sulfate (Sigma-Aldrich), washed 3× in 0.1 M PBS, mounted, dehydrated and sealed with resin (Entellan, Millipore, Billerica, MA, USA).

### Immunofluorescence

For the BrdU immuno-labeling, the brain sections were rinsed three times in 0.1 M PBS and incubated in 2 N HCl at 37°C for 15 min, followed by 0.1 M borate buffer (pH = 8.6) for 10 min and washed 3× with 0.1 M PBS. Sections were incubated in blocking solution for 40 min. Then, sections were incubated with some combinations of the following primary antibodies: rat anti-BrdU (1:500; AbD Serotec Cat # OBT0030, RRID:AB_609568), mouse anti-NeuN (1:500; Millipore Cat # MAB377, RRID:AB_2298772), rabbit anti-GFAP (1:100; Dako Cat # Z0334, RRID:AB_10013382), rabbit anti-Sox2 (1:500, Abcam Cat # AB97959; RRID:AB_2341193), guinea pig anti-doublecortin (DCX, 1:1000: Millipore Cat# AB2253, RRID:AB_1586992) in blocking solution at 4°C overnight. Sections were rinsed 3× with 0.1 M PBS, and incubated in 0.1 M PBS containing 10% fetal bovine serum and conjugated secondary antibodies (Alexa Fluor^®^ 488 anti-rat Cat # A-21208; Alexa Fluor^®^ 594 anti-rat Cat # A-11007; Alexa Fluor^®^ 488 anti-mouse Cat # A32723; Alexa Fluor^®^ 594 anti-rabbit; Alexa Fluor^®^ 594 Cat# R37117; anti-guinea pig Cat# A-11076; dilution 1:1000; Thermo Fisher) for 1 h at room temperature and washed 3× with 0.1 M PBS. Nuclear counterstaining was done with 4′,6-diamidino-2-phenylindole (DAPI; Abcam Cat # ab104139, Cambridge, MA, USA).

### TUNEL Assay

TUNEL staining was performed using the *in situ* cell-death detection kit TMR red (Roche Cat # 12156792910) and following the manufacturer’s directions. Briefly, the sections were fixed on a coverslip, washed 2× with 0.1 M PBS, and then incubated in a permeabilization solution (0.1 M PBS, 0.1% Triton X-100, 0.1% sodium citrate) for 15 min at 4°C. Samples were then washed 2× in 0.1 M PBS and blocked for 10 min in Tris-HCl (pH = 7.5) with 10% fetal bovine serum. Brain tissue was washed 2× and incubated in the TUNEL reaction mixture for 1 h at 37°C in the dark. Nuclear staining was performed with DAPI.

### Quantification

To quantify the number cells per group, brain slices were consecutively numbered, and six sections (taken 120-μm apart) per animal were randomly selected along the rostro-caudal axis. Sectioning interval was from −0.82 mm to −3.70 mm and 0.38 mm to −1.94 mm coordinates relative to Bregma for hippocampus and BC, respectively. We only counted the c-Fos+ cells observed in the same focal plane. Quantifications were made under inverted bright-field microscopy (Zeiss Axio Observer D1, Germany) with the 20× objective (NA = 0.45; area = 0.63 mm^2^ per field). To quantify double labeling, six slices by group were randomly selected in −0.82 mm to −3.70 mm coordinates relative to Bregma (Paxinos and Watson, 2007). For quantification purposes only, the dorsal aspect of the hippocampus was analyzed. Double-labeled cells were quantified only when the DAPI staining colocalized with the cell marker expression and it was confirmed by the orthogonal views. For DCX, Sox2, GFAP cell counting was done in the SGZ along both upper and lower blades of the dorsal aspect of DG. The SGZ was defined as the two-nucleus-wide band below the evident border between the granule cell layer and the hilus. For NeuN, c-Fos, Calbindin and TUNEL, we included the granular zone of the DG. Double-staining quantifications were made with a confocal microscope (Zeiss LSM 700, Germany) with a 40× objective (NA = 1.3; area = 0.15 mm^2^ per field). For confocal analyses, the number of double-labeled cells was quantified in at least 125 single-plane confocal pictures per region (CA1, CA2, CA3 and DG) in each group. To minimize the error for identifying double labeling in a single optical plane, 0.55-μm optical sections were used. To calculate the proliferation rate of SGZ progenitor cells, we determined the percentage of BrdU+ cells expressing a cell identity marker (GFAP, Sox2 or DCX) between the total number of cells expressing that marker. In all cases, these quantitative analyses were done by a researcher blinded to group assignments.

### Densitometry (Relative Optical Density)

Tissue densitometry was used to establish the expression level of calbindin by quantifying the optical density as described previously (Kuchukhidze et al., 2015). Briefly, six brain sections per animal (*n* = 5 mice *per* group) were randomly selected by using the same strategy described above. At least eight pictures per brain section were taken along the whole DG with an inverted microscope (Zeiss Axio Observer D1, Germany) under the 40× objective (NA = 0.95). Images were processed with the software *IMAGEJ 1.46r*^1^. First, each picture was converted to an 8-bit image and the DG was delineated with the section tool of the software. We measured area integrated density and mean gray value in every antibody-treated section. Negative controls (with the omission of incubation with primary antibody) were included for each brain section analyzed. We calculated the corrected total marker expression with the following formula: Corrected marker expression = Integrated density (selected area × the mean intensity of background staining; McCloy et al., 2014). A blinded researcher to group assignment performed the densitometry measurement and calculations.

### Statistical Analysis

We used the Shapiro-Wilk test to determine if the data were normally distributed and applied the appropriate statistical test to establish statistical differences. For the statistical analysis of histological quantifications, we used the Mann–Whitney “*U”* test. To determine intra group differences in the behavioral test we utilized Friedman’s test, whereas intergroup differences (control vs. experimental) were calculated with the Mann-Whitney “*U*” test. *Chi*-square test was used for the analysis of the search strategies of memory test. Data are expressed as median (Mdn) and interquartile range (IQR). The level of confidence was set at 95% (*p* < 0.05).

## Results

### Whisker Fulguration Decreases Cytochrome C Oxidase and c-Fos-Expressing Cells in Barrel Cortex

**Figure 1:**
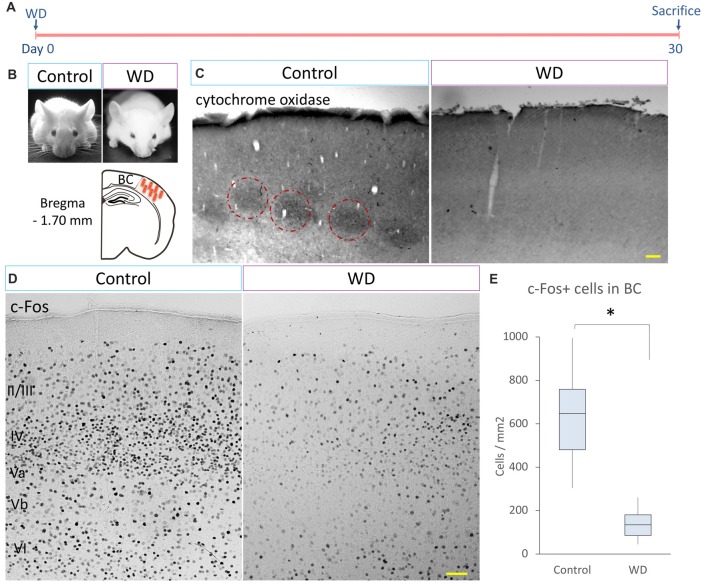
Cytochrome C oxidase activity and c-Fos-expressing cells in the barrel cortex (BC). **(A)** Experimental design. **(B)** CD-1 mice 30 days after surgical manipulation (sham or whisker deprivation (WD)); below: representative coronal section at −1.70 mm coordinates relative to Bregma. **(C)** The control group shows the typical barrel pattern as identified by the cytochrome C activity staining (dotted circles). Note that the whisker-deprived (WD) group did not show cytochrome C activity staining. **(D)** Immunostaining for c-Fos (black dots) in the BC in controls and the WD group. **(E)** Quantification of c-Fos+ cells in the BC. The WD group showed a decrease in the density of c-Fos-expressing cells as compared to controls. Data are expressed as median and interquartile range (IQR). *U = 0, *p* < 0.001; Mann-Whitney “*U*” test; *n* = 5 animals per group. Bars = 50 μm.

c-Fos protein is coded by a calcium-dependent immediate early gene that is used as a marker of neuronal activity (Herrera and Robertson, 1996). Tactile experience and whisker stimulation increase c-Fos expression (Filipkowski et al., 2000), whereas whisker trimming reduces c-Fos expression in the BC (Filipkowski et al., 2001). To determine changes in the neuronal activity that occur after permanent WD, we fulgurated whiskers in P60 mice and analyzed the expression of cytochrome oxidase and c-Fos (*n* = 5 mice *per* group). At day 30 after WD, we stained brain sections to detect the activity of cytochrome C oxidase in the BC and found that the control group showed the typical barrel distribution (Wong-Riley and Welt, 1980), which was absent in the WD group (Figures 1A,B,D). We then quantified the density of c-Fos+ cells in the BC and observed that the WD group had a dramatic reduction in the density of c-Fos+ cells (Mdn = 104 cells/mm^2^ of BC region, IQR: 66–139) as compared with the control group (Mdn = 497 cells/mm^2^ of BC region, IQR: 381–597; *U* = 0, *p* < 0.001; Figures 1C,E). These findings indicate that whisker fulguration produces a persistent reduction in neural activity of the BC in the adult brain.

### Long-Term WD Reduces the Density of c-Fos+ Cells and Calbindin in the Hippocampus

**Figure 2:**
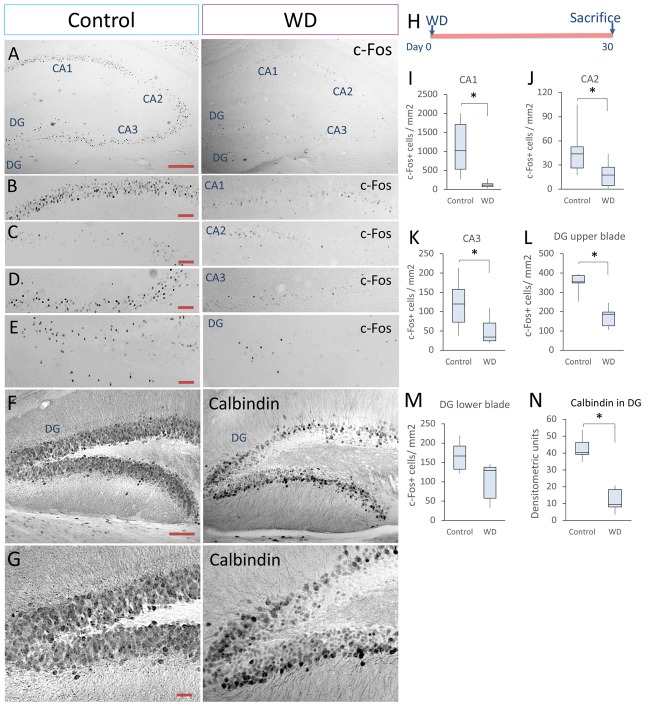
Whisker elimination decreases the density of c-Fos+ cells and calbindin expression in the hippocampus.** (A)** Immunostaining of c-Fos+ cells in the hippocampus of both groups (black dots). **(B–E)** Higher magnifications of CA1, CA2, CA3 and dentate gyrus (DG). **(F)** Immunostaining of calbindin-D_28k_ in the hippocampus of both groups. **(G)** High magnifications of calbindin expression in DG. **(H)** Experimental design. **(I–L)** Quantification data of the c-Fos+ cells in CA1 **(I)**, CA2 **(J)**, CA3 **(K)**, upper blade of DG **(L)** and lower blade of DG **(M)**. **(N)** Quantification of the relative optical density of calbindin immunostaining in both groups. Data are expressed as median and IQR; *n* = 5 mice per group; *P < 0.01; Mann-Whitney “*U*” test. Bars **(A,F)** = 50 μm. Bars **(B–E)** = 25 μm. Bar **(G)** = 15 μm.

Adult hippocampus creates spatial maps by integrating multiple neural inputs from visual, olfactive and tactile clues (Pereira et al., 2007; Haggerty and Ji, 2015; Zhang and Manahan-Vaughan, 2015). Whisker information is partially processed in the hippocampus during texture discrimination tasks and memory integration (Grion et al., 2016). To determine whether permanent WD modified the neuronal activity in the adult hippocampus, we extended our analysis of the c-Fos-expressing cells to CA1, CA2 and CA3 regions. Our data indicated that WD produced a ~4-fold reduction in the density of c-Fos+ neurons in the hippocampal regions (Figures 2A–D,H–K): CA1 (controls = 1020 cells/mm^2^ of CA1 region, IQR: 531–1706 vs. WD = 113 cells/mm^2^ of CA1 region, IQR: 73–151; *U* = 22.5, *p* < 0.001) CA2 (controls = 43 cells/mm^2^ of CA2 region, IQR: 26–52 vs. WD = 18 cells/mm^2^ of CA2 region, IQR: 4–35; *U* = 73.5, *p* < 0.001), and CA3 (controls = 119 cells/mm^2^ of CA3 region, IQR: 70–157 vs. WD = 34 cells/mm^2^ of CA3 region, IQR: 24–70; *U* = 125.5, *p* < 0.001). Remarkably, the density of c-Fos+ cells was significantly reduced in the DG (Figures 2E,L,M), a neurogenic region that importantly contributes to neural plasticity (Sahay et al., 2011). The upper blade of DG showed a significant decrease in the density of c-Fos+ cells in the WD group (Mdn = 186 cells/mm^2^ of upper blade region, IQR: 127–197) as compared to the control group (Mdn = 354 cells/mm^2^ of upper blade region, IQR: 348–387; *U* = 0, *p* = 0.009). No statistically significant differences were found in the lower blade of DG: Controls (Mdn = 166 cells/mm^2^ of lower blade region, IQR: 132–192) vs. WD animals (Mdn = 129 cells/mm^2^ of lower blade region, IQR: 57–137; *U* = 5, *p* = 0.11). These findings indicate that permanent WD reduces the density of c-Fos+ cells, a calcium-dependent early gene product in the DG. To determine whether local calcium homeostasis was also altered by WD, we analyzed the expression of another calcium-dependent protein, the calcium-binding protein calbindin-D_28k_, in the DG (Figures 2F,G,N). We observed that calbindin expression in granule cells was dramatically reduced in the WD group (Mdn = 9.5 densitometry units IQR: 7.9–18.2) as compared to the control group (Mdn = 40.2 densitometry units IQR: 39.1–46.3; *U* = 0, *P* < 0.0001). Taken together, our findings indicate that permanent vibrissal deprivation produces a sustained reduction in c-Fos and calbindin in the adult hippocampus, including the DG.

### WD Reduces the Proliferation of Neural Progenitor Cells in the Hippocampus

**Figure 3:**
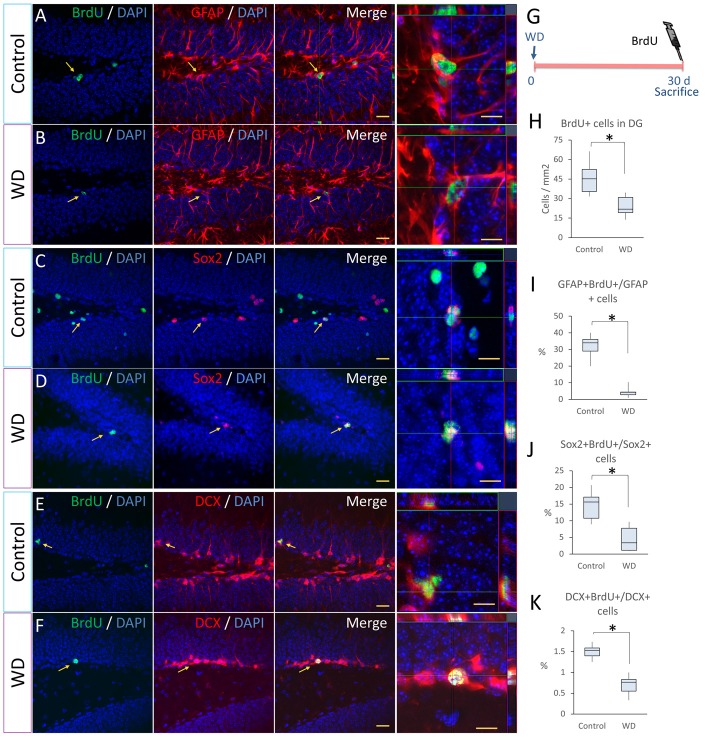
Whisker elimination reduces the proliferation rate of primary progenitor cells and neuroblasts in the subgranular zone (SGZ). **(A–F)** Immunostaining of Bromodeoxyuridine (BrdU)+ (green)/GFAP+ (red), BrdU+/Sox+ (red) and BrdU+/doublecortin (DCX)+ (red) in control and WD groups. **(G)** Experimental design: at day 30 after WD, 50 mg/kg BrdU was injected intraperitoneally 2 h before sacrifice to determine the proliferation of neuronal progenitors. **(H)** Quantification of BrdU+ cells in the DG. **(I)** Percentage of GFAP+BrdU+/GFAP+ cells. **(J)** Percentage of Sox2+BrdU+/Sox2+ cells. **(K)** Percentage of DCX+BrdU+/DCX+ cells. All nuclei were stained with 4′,6-diamidino-2-phenylindole (DAPI; blue). Data are expressed as median and IQR. Insets show high magnification views. *n* = 5 animals per group; *P < 0.05; Mann-Whitney “*U*” test. Bar **(A–F)** = 30 μm. Bar in orthogonal views = 10 μm. Optical section thickness = 0.55 μm.

The SGZ of DG is a discrete brain region that produces new neurons throughout life (Gonçalves et al., 2016). Activity-dependent inputs and calcium homeostasis modulate adult neurogenesis (Kempermann et al., 1997; Palop et al., 2003; Pereira et al., 2007; Ma et al., 2009). Our data indicated that the calcium-dependent neuronal activity in the DG is reduced by the effect of WD. These alterations may be due to functional variations (less neuronal activity) or structural changes (less neuronal production). To determine whether the whisker elimination affected the neuronal production in the SGZ, we injected BrdU 2 h before sacrifice (*n* = 5 mice *per* group) and studied the proliferation rate of hippocampal neural progenitors (Soto-Rodriguez et al., 2016; Figures 3A–K). We quantified the number of BrdU+ cells in the SGZ and found a statistically significant reduction in WD mice (Mdn = 21.8 cells/mm^2^ of SGZ area, IQR: 19.2–30.9) as compared with the control group (Mdn = 45.2 cells/mm^2^ of SGZ area, IQR: 35.4–52.4; *U* = 6, *P* = 0.001; Figure 3H). We then investigated by confocal microscopy if some of these BrdU+ cells corresponded to hippocampal neural progenitors, i.e., radial-glia-like GFAP+ cells as confirmed by the presence of a pyramidal soma with a long vertical process extending from the SGZ towards the molecular layer and branching, as well as small horizontally oriented processes along the SGZ (Kosaka and Hama, 1986; Seri et al., 2001; Kronenberg et al., 2003; Steiner et al., 2006). We found that the WD group suffered a ~3-fold decrease in the number of BrdU+GFAP+ cells with radial-glia morphology (Mdn = 3.7 cells/mm^2^ of SGZ area, IQR: 3.2–4.3) as compared with controls (Mdn = 12.6 cells/mm^2^ of SGZ area, IQR: 10.8–13.5; *U* = 0; *P* = 0.004). We then calculated the proliferation rate of radial-glia-like GFAP+ cells (Figures 3A,B,I). Our data indicate that the proliferation rate of radial-glia-like GFAP+ cells showed a statistically significant decrease in the WD group (Mdn = 10%, IQR: 8.5–10.1) as compared to controls (Mdn = 34%, IQR: 29–36; *U* = 0; *P* = 0.004).

To establish whether WD reduces the proliferation of other hippocampal progenitor cells (Kriegstein and Alvarez-Buylla, 2009; Gonçalves et al., 2016), we analyzed the expression of the Sox2 transcription factor in the SGZ (Figures 3C,D). Our data indicated that the WD group had a significant decrease in the number of BrdU+Sox2+ cells (Mdn = 1.5 cells/mm^2^ of SGZ area, IQR: 1.5–5.5) respect to controls (Mdn = 28.5 cells/mm^2^ of SGZ area, IQR: 20.2–37.3; *U* = 0, *p* = 0.004). We calculated the proliferation rate of Sox2+ progenitor cells (Figure 3J) and found that the WD group had a significant reduction in the percentage of Sox2+BrdU+/Sox2+ cells (Mdn = 3.3%, IQR: 1.1–7.7) when compared to the control group (Mdn = 15.6%, IQR: 10.7–17.1; *U* = 3; *P* = 0.016). Since these findings suggested that WD can affect the pool of radial-glia-like GFAP+ cells and Sox2-expressing progenitor cells, we decided to determine the percentage of Sox2+ cells that co-express GFAP with radial-glia-like morphology in the SGZ. Interestingly, we did not find statistically significant differences in the percentage of Sox2+GFAP+ radial-glia-like astrocytes between the WD group (Mdn = 18% IQR: 16.2–21.75%) and the control group (Mdn = 23% IQR: 20.2–27.7%; *U* = 25, *P* = 0.065). This evidence suggests that the reduction in the proliferation of Sox2+ cells found in the WD group is not due to a decrease in the density of radial-glia-like GFAP+ astrocytes.

To determinate whether the permanent WD affected the proliferation of neuroblasts in the SGZ (Song et al., 2012), we co-stained sections with BrdU and a neuroblast marker DCX and quantified the number of BrdU/DCX co-expressing cells in the SGZ (Figures 3E,F). We observed an important decrease in the number of co-labeled cells in WD animals (Mdn = 2.3 cells/mm^2^ of SGZ area, IQR: 1.7–2.6) when compared to controls (Mdn = 6.9 cells/mm^2^ of SGZ area, IQR: 5.2–7.5; *U* = 0, *p* = 0.005). We also calculated the proliferation rate of DCX+ cells (Figure 3K) and found that the WD group showed a significant reduction in the percentage of DCX+BrdU+/DCX+ cells (Mdn = 0.76%, IQR: 0.55–0.83) when compared to the control group (Mdn = 1.54%, IQR: 1.41–1.6; *U* = 0; *P* = 0.004). We then quantified the absolute number of neuroblasts found in both groups, we observed that the number of neuroblasts was significantly reduced by the whisker removal: WD mice (Mdn = 181 cells cells/mm^2^ of SGZ area, IQR: 162–197) vs. controls (Mdn = 441 cells/mm^2^ of SGZ area, IQR: 419–556; *U* = 0, *p* = 0.004). Taken together, these findings indicate that the permanent loss of tactile inputs from whiskers substantially affects the different aspect of the neurogenic process in the adult SGZ.

### WD Affects the Proliferation and Maturation and Promotes Apoptosis in Dentate Gyrus

**Figure 4:**
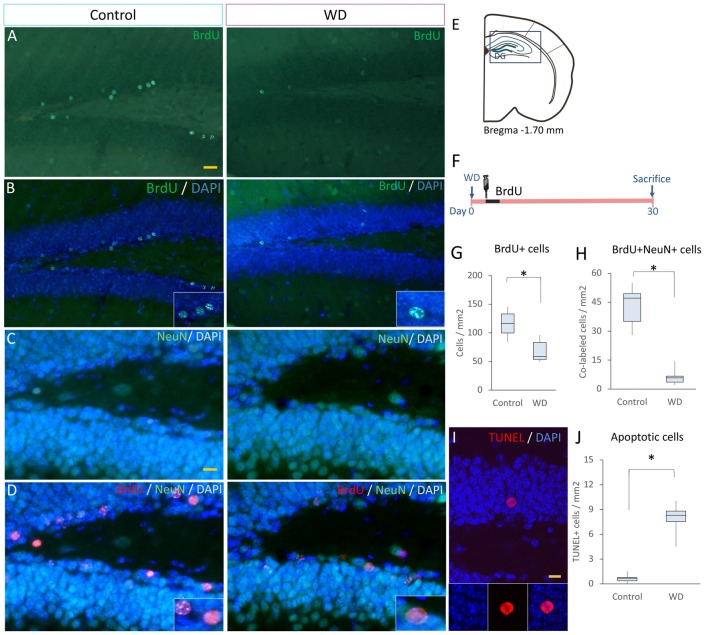
The final number of newly generated neurons is decreased in whisker-deprived animals. **(A,B)** Immunostaining for BrdU (green) in the control and the whisker-deprived (WD) group. **(C,D)** Double immunostaining of NeuN+ (green)/BrdU+ (red). **(E)** A representative coronal section at −1.70 mm coordinate relative to Bregma. **(F)** Experimental design: 3 weeks after the last BrdU injection (50 mg kg^−1^BrdU/8 h i.p. for 3 days), we determined the number of NeuN+ and BrdU+ cells. **(G)** BrdU+ cells in the DG, the analysis showed less BrdU+ cells in the WD group. **(H)** Quantification of BrdU+NeuN+ in the DG. **(I)** Confocal photography of a TUNEL positive cell and the results of TUNEL quantification in DG **(J)**. All nuclei were stained with DAPI (blue). Data are expressed as median and IQR. Insets: high magnification views. *n* = 5 per group; *P < 0.001; Mann-Whitney “*U*” test. Bar **(A,B)** = 20 μm; Bar **(C,D)** = 10 μm; Bar **(I)** = 10 μm. Optical section thickness = 0.55 μm.

Our data indicated that WD affects the proliferation of SGZ progenitors. This event may affect the ultimate number of new-born neurons in the DG. To investigate this possibility, at the 3rd day after whisker removal, we injected BrdU every 8 h for 3 days and sacrificed these mice 30 days later (*n* = 5 *per* group). We found that the number of BrdU+ cells that remained in the DG of WD group declined by approximately ~3-fold (Mdn = 58 cells/mm^2^ of DG area, IQR: 53–83) with respect to the control group (Mdn = 116 cells/mm^2^ of DG area, IQR: 99–133; *U* = 2, *P* = 0.011; Figures 4A,B,E–G). To establish the number of BrdU+ cells that corresponded to NeuN+ mature neurons (Kempermann et al., 2015), we co-stained the sections with anti-NeuN antibodies and quantified BrdU/NeuN co-labeled cells. We found that the number of BrdU+NeuN+ cells in the control group (Mdn = 44 cells/mm^2^ of DG area, IQR: 33–53) was significantly higher than that in the WD group (Mdn = 10 cells/mm^2^ of DG area, IQR: 8–11; *U* = 0, *P* = 0.002; Figures 4C,D,H). These findings suggest that the cell survival of newborn neurons or a reduction in cell proliferation of precursor cells in the DG may be affected by the WD. We then estimated the percentage of BrdU+NeuN+/BrdU+ cells in the DG, our data indicated that the WD group showed a significant reduction in the percentage of co-labeled cells as compared to controls (WD: 17.7%, IQR 10.5–22.5% vs. controls 40.4%, IQR 39.3–40.9%; *U* = 0, *P* = 0.004). These findings suggest there is an alteration in the final proportion of new neurons produced in the hippocampus of WD animals. Thus, we investigate whether this reduction in adult neurogenesis was partially due to a higher apoptosis rate. We analyzed the number of apoptotic cells (TUNEL+ cells; Figures 4I,J) in the DG (*n* = 5 mice *per* group). Our results showed that the WD group had ~10-fold more apoptotic cells (Mdn = 8.2 cells/mm^2^ of DG area, IQR: 7.5–8.7) as compared to the control group (Mdn = 0.7 cells/mm^2^ of DG area, IQR: 0.4–0.75; *U* = 0, *P* = 0.009). Altogether, our findings indicate that permanent tactile deprivation significantly reduces the production of newly-generated neurons in the DG.

### WD Affects the Spatial Memory

**Figure 5:**
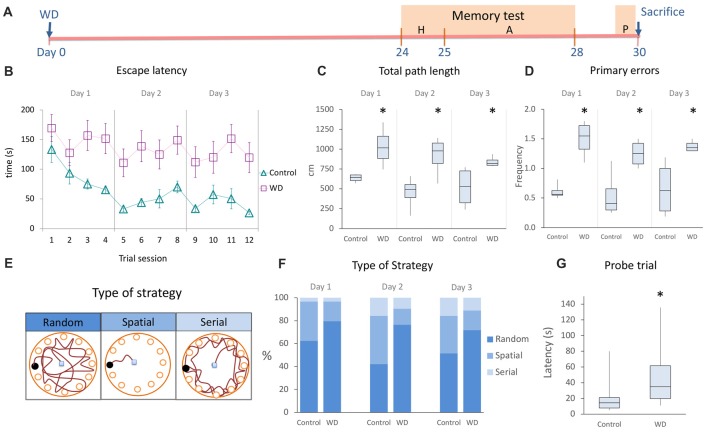
The whisker elimination impairs the acquisition and retention of spatial memory in the Barnes Maze.** (A)** Experimental design of memory test. **(B)** Spatial memory acquisition by trial, escape latency represents the time employed to find the goal box. **(C)** Total path length (distance traveled by mice for the entire duration of assays). **(D)** Primary errors (number of errors to the first encounter of the escape hole). **(E)** Schematic drawings of the type of strategy used to solve the Barnes Maze. **(F)** Percentage of type of strategy used to find the escape hole during the acquisition period. Controls preferentially used the spatial strategy as compared to WD (*χ^2^* = 4.94, *p* = 0.026), whereas WD mice solve the task by using more frequently the random strategy than controls (*χ*^2^ = 6.433, *p* = 0.011). **(G)** Probe trial: the WD group required more time to find the target hole. Habituation phase (H); memory acquisition phase (A); Probe trial (P). In the learning curve, data are expressed as mean ± SEM. The other plots show median and IQR; *n* = 16 per group. *P < 0.05; Mann-Whitney “*U*” test.

Visual information and the head-direction system are required for spatial learning (Knierim et al., 1995; Dombeck et al., 2010; Youngstrom and Strowbridge, 2012). While the role of these systems in acquisition of hippocampal-dependent memory has been extensively studied, the role of vibrissal system during spatial learning is not clear. To establish whether the hippocampal alterations induced by WD can affect spatial memory performance, we used the Barnes maze paradigm 24 days after WD to evaluate hippocampal-dependent memory (Figures 5A–E). We first analyzed whether animals learned the task by determining intragroup differences in the time spent to reach the goal box. Our data indicated that both controls (*χ^2^* = 5.33 s, *p* = 0.021, Friedman’s test) and WD animals (*χ*^2^ = 9 s, *p* = 0.003, Friedman’s test) efficiently learned the task. Then, we examined intergroup differences and observed that the control group showed a continuous reduction in escape latency during the whole acquisition phase as compared to the WD group: day 1 (controls = 67 s, IQR: 39–122 vs. WD mice = 197 s, IQR: 44–240; *U* = 1420, *p* = 0.003); day 2 (controls = 34 s, IQR: 20–56 vs. WD mice = 106 s, IQR: 41–240; *U* = 1048, *p* < 0.001); day 3 (controls = 27 s, IQR: 18–45 vs. WD mice = 70 s, IQR: 26–240; *U* = 1113, *p* < 0.001; Figure 5B). We also recorded the total path length and the number of primary errors, two parameters indicative of spatial learning (Harrison et al., 2006). The control group presented path lengths shorter than those presented by the experimental group (Figure 5C): day 1 (controls = 643 cm, IQR: 604–674 vs. WD mice = 1016 cm, IQR: 881–1164; *U* = 0, *p* = 0.021); day 2 (controls = 491 cm, IQR: 388–531 vs. WD mice = 978 cm, IQR: 841–1076; *U* = 0, *p* = 0.021); day 3 (controls = 531 cm, IQR: 323–726 vs. WD mice = 818 cm, IQR: 795–862; *U* = 0, *p* = 0.021). Consistently, control animals also made significantly fewer primary errors than WD mice (Figure 5D): Day 1(WD = 1.6 errors, IQR: 1.3–1.7 vs. controls 0.6 errors, IQR: 0.5–0.6; *U* = 0, *p* = 0.02); day 2 (WD = 1.3 errors, IQR: 1–1.4 vs. controls 0.4 errors, IQR: 0.3–0.6; *U* = 0, *p* = 0.021); day 3 (WD = 1.4 errors, IQR: 1.3–1.45 vs. controls 0.6 errors, IQR: 0.28–0.6; *U* = 0, *p* = 0.02). Taken together, these data suggest that WD mice can learn, but their memory acquisition is deficient.

During the acquisition phase, we observed that our mice used different search strategies to solve the task. Hence, we analyzed the frequency of three well-known behavioral strategies: random, serial and spatial (Jašarević et al., 2011; Figures 5E,F). Our findings indicate that the spatial strategy (hippocampal-dependent task) was more frequently used by the control group (day 1 = 34.34%, day 2 = 42.2%, and day 3 = 34.4%) than WD animals (day 1 = 15.6%, day 2 = 14%, and day 3 = 17.1%; *χ^2^* = 4.94, *p* = 0.026). In contrast, the random strategy (non-hippocampal-dependent task) was more frequently used by the WD group (day 1 = 81.3%, day 2 = 76.6%, and day 3 = 73.4%) than control animals (day 1 = 62.5%, day 2 = 42.2%, and day 3 = 51.6%; *χ*^2^ = 6.433, *p* = 0.011). No statistically significant differences were found for the use of serial strategy (*χ*^2^ = 0.286, *p* = 0.593). Taken together, these data indicate that WD animals tend to solve the behavioral paradigm with a non-hippocampal dependent strategy.

Our previous findings indicate that WD alters memory acquisition. To investigate whether the memory retention was also affected by whisker elimination, we repeated the behavioral task 48 h after the last trial (Figure 5G). We observed that WD animals needed more time to find the goal hole (Mdn = 35 s, IQR: 19.7–61.5) than controls (Mdn = 14.5 s, IQR: 7.7–21; *U* = 47, *p* = 0.002). Altogether, our data indicate that WD significantly impairs the hippocampal-dependent memory acquisition and retention.

## Discussion

**Figure 6:**
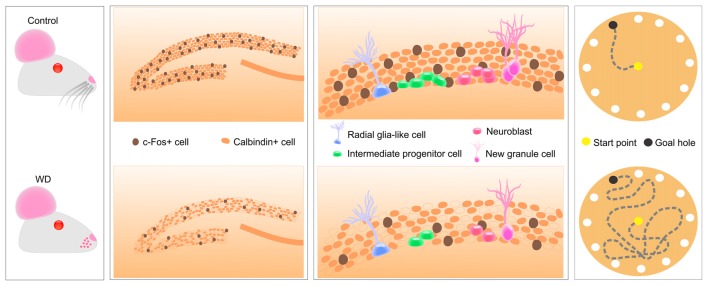
Tactile information associated with fine whisker discrimination is a strong regulator of hippocampal activity that changes the expression of calcium-dependent proteins in DG, disrupts several aspects of hippocampal neurogenesis and impairs spatial memory acquisition and retention.

In the present study, we produced tactile deprivation by cauterizing whisker follicles and evaluated whether these sensorial inputs can modify the hippocampal function. Our findings indicated that, 30 days after whisker removal, the cytochrome oxidase activity in the BC remains absent. This enzymatic activity reduction is associated with approximately 80% reduction in the density of c-Fos-expressing cells of the BC and the hippocampal CA1, CA2, and CA3 regions. Remarkably, the expression of c-Fos and calbindin in the DG was profoundly affected by whisker elimination. Thus, our next goal was to evaluate whether the low expression of these calcium-dependent proteins could be due to a decline in the hippocampal neurogenesis. Strikingly, we found that whisker elimination dramatically reduces the cell proliferation of neurogenic progenitors in the SGZ. We also observed that these histological abnormalities coincided with a defect in the hippocampal-dependent memory. Altogether, these findings indicate tactile deprivation reduces neuronal activity and some calcium-dependent proteins in the hippocampus, disrupts the hippocampal neurogenesis and impairs spatial memory (Figure 6).

Rodents need whiskers to acquire tactile information from the environment. These tactile inputs are processed in the BC, at layer IV of the somatosensory cortex (Petersen, 2007). We produced a chronic tactile deprivation by fulgurating the vibrissal follicles as previously described (Fetter-Pruneda et al., 2013) and used the expression of cytochrome oxidase to label the BC (Wong-Riley and Welt, 1980). At day 30 after WD, the expression of cytochrome oxidase was not observed in the BC, indicating that tactile inputs were permanently disrupted by our method. We then evaluated the number of neurons expressing the c-Fos protein, a calcium-dependent immediate early proto-oncogene that is triggered by a different stimulus and may change the structural and functional properties of neural cells (Miller et al., 1984; Herrera and Robertson, 1996). Our data indicated that the expression of c-Fos in the BC is dramatically reduced by whisker elimination. This evidence confirmed that tactile disruption produces a long-term reduction in the density of c-Fos expressing cells. Short- and medium-term reductions in the c-Fos expression has been described with other models of tactile deprivation, such as: whisker plucking (Kaliszewska et al., 2012), whisker clipping (Filipkowski et al., 2001; Bisler et al., 2002) and whisker trimming (Staiger et al., 2002). In contrast, the c-Fos expression level in somatosensory cortex is increased by animal exposure to enriched environments or a direct stimulation of neural tissue (Mack and Mack, 1992; Filipkowski et al., 2000).

Electrical stimulation of the infraorbital nerve (that carries information from whiskers) and touch-guided behavior evoke neuronal firing in the CA1 hippocampal region (Pereira et al., 2007; Itskov et al., 2011). These tactile inputs increase the activity of CA1 hippocampal neurons the CA1 region through thalamocortical relays and are associated with fine sensorial discrimination (Pereira et al., 2007). During early brain development, WD reduces the activity of CA3 neurons that induces CA3-CA1 synaptic facilitation (Milshtein-Parush et al., 2017). For this reason, we decided to evaluate c-Fos expression in four hippocampal regions: CA1, CA2, CA3 and DG. Our findings indicate that long-lasting tactile deprivation reduces in approximately 80% the density of c-Fos+ cells in all these regions. Interestingly, we observed that c-Fos reduction was statistically significant only in the upper blade of DG in WD animals. The suprapyramidal (upper) blade, more than the infrapyramidal (lower) blade, is strongly activated by novel environment exploration (Guenthner et al., 2013). Therefore, our findings suggest that whisker activity triggered by environment exploration/navigation is modulating the neuronal activity in the suprapyramidal blade of DG.

We also found a significant reduction of the calcium-binding protein calbindin-D_28k_ in granule cells of the DG, which suggests that calcium homeostasis is strongly affected by permanent WD. Our experimental approach does not allow us to establish the biomolecular mechanism of these changes, but we hypothesize that WD may provoke hyper-excitability of hippocampal neurons as a homeostatic mechanism for sensorial deprivation. This kind of compensatory phenomenon has been previously reported and appears to be related to hypersensitivity to sensory inputs in the adult hippocampus (Zhang and Manahan-Vaughan, 2015). Interestingly, neuronal depletion of calcium-dependent proteins in the DG has also been tightly linked to cognitive impairment, neurodegeneration, aging and Alzheimer’s disease (Palop et al., 2003; Moreno et al., 2012; Kook et al., 2014). However, we do not know whether the neuronal depletion of calcium-dependent proteins that follows permanent tactile deprivation is a predisposing condition for psychiatric disorders, cognitive decline and/or neurodegeneration. Our evidence indicates that whisker inputs may play an important role in neural plasticity, calcium homeostasis and synaptic connectivity of the adult hippocampus as that observed in cortical regions (Kaliszewska et al., 2012; Mangin et al., 2012; Pignataro et al., 2015).

The DG is a neurogenic region that produces new neurons in the adult brain throughout life. This specialized niche contains neural stem cells that originate neuroblasts, which migrate to the adjacent granular layer and spread their axons to local circuits in the adult hippocampus (Kempermann et al., 2015). Inputs from voluntary exercise, enriched environments, cognitive and emotional processes comprise the activity-dependent control of hippocampal functions that, in turn, modulates the adult neurogenesis (Kempermann et al., 1997; Pereira et al., 2007; Ma et al., 2009). Our results showed an important reduction in the density of c-Fos+ cells and calbindin expression in the DG after whisker elimination. Thus, we decided to investigate whether the long-term reduction in calbindin and c-Fos might affect the proliferation of primary neural progenitors (GFAP+ and Sox2+ cells) in the DG (Suh et al., 2007). Strikingly, WD animals show less number of BrdU+ neural progenitors. This reduction in the number of BrdU+Sox2+ cells was not due to a reduction in the pool of radial GFAP+ cells as indicated by the absent of significant changes in the number of Sox2+GFAP+ radial cells between groups. To verify if these cellular changes modified the final pool of new neurons that are continuously incorporated into the DG, we injected BrdU at day three and sacrificed the animals 30 days after whisker elimination. Strikingly, we found that the number of newly-generated mature neurons (NeuN+BrdU+) was reduced by 50%. This reduction was associated with an increase in the number of detectable apoptotic cells (TUNEL+ cells) in the WD group. Therefore, our data indicate that tactile information processed in the hippocampus regulates the addition of newly-generated neurons by reducing the proliferation of neural progenitor cells in the DG. WD induces presynaptic inhibition in CA3 and changes in the AMPA-mediated synaptic transmission (Milshtein-Parush et al., 2017). These changes are associated with a reduction in the AMPA/NMDA ratio and an increase in NR2B-containing NMDA receptors (Milshtein-Parush et al., 2017). Interestingly, *in vivo* administration of the NMDA receptor agonists reduce proliferation, whereas NMDA-receptor antagonists promote the proliferation of neural progenitor cells in the DG (Cameron et al., 1995; Kitayama et al., 2003; Halim et al., 2004). Therefore, this evidence excitingly suggests that WD may regulate the proliferation of hippocampal progenitor cells by maintaining the neurotransmitter balance in the adult hippocampus.

The adult hippocampus is a crucial region for the acquisition of spatial memory and navigation (Morris et al., 1982). The DG is a hippocampal region that regulates pattern separation, which helps distinguish similarly encoded contextual information (McHugh et al., 2007). Many studies have linked visual, self-motion and vestibular clues to spatial learning (Knierim et al., 1995; Dombeck et al., 2010; Youngstrom and Strowbridge, 2012), suggesting that spatial memories can be formed using visual information and the head-direction system. While spatial learning has been extensively studied, the role of vibrissal system during spatial learning was unclear. A previous report suggested that vibrissae trimming affected only the proprioceptive location of the escape platform in the Morris maze, without affecting memory acquisition (Grigoryan et al., 2005). Additionally, pharmacologically induced vibrissae paralysis did not affect the animal’s ability to learn (Patarroyo et al., 2017). We used the Barnes maze to evaluate both spatial memory and navigation (Barnes, 1979; Soto-Rodriguez et al., 2016). Our data indicated that the permanent deprivation of whiskers notably impairs the acquisition of spatial memory, as shown by longer latencies spent to find the goal holes. A possible explanation for some inconsistent findings between Morris and Barnes mazes in WD models may be due to the solving strategy for the Barnes maze requires both visual and tactile skills (Barnes, 1979; Soto-Rodriguez et al., 2016). Thus, Barnes may be a more sensitive behavioral paradigm to evaluate memory integration that is required to create spatial maps (Grion et al., 2016). Our data also indicated that 48 h after this acquisition deficit had a negative impact on memory retention in the whisker-deprived animals, which indicates a poor learning performance. Remarkably, whisker-deprived animals tend to solve the maze by using a random-based strategy, non-hippocampal dependent task (Harrison et al., 2006), more frequently than controls, while the control group preferentially used a spatial-based strategy, a hippocampus-dependent task, to solve the maze. Altogether, our findings indicate that whisker information is very important to create spatial memory and execute spatial navigation. Thus, our evidence strongly suggests that inputs from the tactile system and other sensorial modalities are integrated by hippocampus to form novel spatial memories. Nevertheless, it would be important to investigate whether these events can be reversed or compensated by whisker stimulation and whether partial whisker removal may also have significant consequences in the hippocampal cellularity and function.

## Conclusion

Vibrissal fulguration is an efficient method of permanently removing facial whiskers and analyzing the effects of sensorial deprivation in the adult brain. Taken together, our findings indicate that tactile information associated with fine whisker discrimination is a strong regulator of hippocampal activity that changes the expression of calcium-dependent proteins, alters hippocampal neurogenesis and impairs spatial memory. These findings unveil the neurophysiological interactions among tactile information with the hippocampal neurogenesis and the creation of spatial maps in the adult brain.

## Author Contributions

OG-P: conception and design of experiments, data collection, analysis, interpretation, manuscript writing, final approval of manuscript and financial support. VL-V: data collection, analysis, interpretation and manuscript writing. NI-C: data collection, analysis and interpretation.

## Conflict of Interest Statement

The authors declare that the research was conducted in the absence of any commercial or financial relationships that could be construed as a potential conflict of interest.

## Footnotes

^1^http://imagej.nih.gov/ij/
